# Performance of Different Echocardiographic Measurements of Left Atrial Size in Dogs by Observers with Different Levels of Experience

**DOI:** 10.3390/ani12050625

**Published:** 2022-03-01

**Authors:** Alexander M. Safian, Giulio Menciotti, Sunshine M. Lahmers, Hyeon Jeong, Alessandra Franchini, Michele Borgarelli

**Affiliations:** Department of Small Animal Clinical Sciences, Virginia-Maryland College of Veterinary Medicine, Virginia Polytechnic Institute and State University, 204 Duck Pond Drive, Blacksburg, VA 24061-0442, USA; alexsafian805@vt.edu (A.M.S.); slahmers@vt.edu (S.M.L.); jeonghw@vt.edu (H.J.); alessf9@vt.edu (A.F.); micheb1@vt.edu (M.B.)

**Keywords:** ultrasound, heart, canine, variability, agreement

## Abstract

**Simple Summary:**

Assessing enlargement of the left atrium (one of the four cardiac chambers) is extremely important for gaining information about dogs’ heart disease, their prognosis, and directing treatment. However, people with different levels of experience may be required to make this assessment, and we don’t know how observers with different experiences perform in making this assessment. In this study, five observers with different levels of experience evaluated the left atrium of 36 dogs in a blinded fashion (i.e., unaware of each other measurements, or of the identity of the dog), compared to two cardiologists. We then used statistical analysis to evaluate repeatability, reproducibility, accuracy of the measurements, and the capacity of correctly identifying left atrial enlargement. We found that the measurements performed by observers with more experience where more similar to the cardiologists’ measurements, and that combining more than one technique for measuring the left atrium can improve accuracy of the identification of left atrial enlargement.

**Abstract:**

Assessment of left atrial (LA) sizes in dogs informs clinical staging, risk assessment, treatment decisions, and prognosis. The objective of this study was to assess the diagnostic performance of observers with different levels of experience measuring the LA with three different techniques. Echocardiographic images from 36 dogs with different degrees of left atrial enlargement (LAE) were retrospectively retrieved, anonymized and measured in a blinded fashion by a veterinary student, a first-year cardiology resident, a third-year cardiology resident, and two board-certified veterinary cardiologists. The LA to aortic root ratio (LA:Ao), LA antero-postero diameter indexed to body weight (LAiAPD) and left atrial area were measured. Inter- and intra-observer intraclass correlation coefficients (ICCs) were calculated for all three variables. Bland–Altman plots and accuracy in identification of LAE were calculated for the three least experienced observers using LA:Ao and LAiAPD. Intra- and interobserver ICCs were greater than 0.9 for every variable. The observer with least experience had significant positive bias and a tendency to overestimate larger measurements using LA:Ao, but not using LAiAPD. The accuracy of identification of LAE also increased with the increasing level of experience and was higher for LAiAPD compared to LA:Ao. Combining both methods for identification of LAE, further increased accuracy.

## 1. Introduction

Evaluation of left atrial (LA) sizes in dogs is important for clinical staging, risk assessment, treatment decisions, and prognosis [1,2,3,4,5]. Indeed, published veterinary literature consistently identified this echocardiographic variable as predictive of prognosis in dogs with cardiac diseases [1,5,6,7,8,9]. Furthermore, LA size is fundamental in the staging and assessment of many acquired and congenital canine heart diseases, with LA enlargement (LAE) often used as an indicator of hemodynamic significance [9,10]. Consequently, the evaluation of LA sizes is also key in directing medical treatment for the most common canine heart disease, both in the acute and chronic phases [2,10,11]. Lastly, evaluation of LA sizes is recommended as part of both humans’ and canines’ ultrasonographic assessment of critical patients [12,13].

With the increased availability and use of ultrasonography not only in cardiology specialty practice, but also general practice and emergency settings, the measurement of LA sizes and identification of patients with left atrial enlargement (LAE) is often performed by observers with different level of experience. However, the repeatability and reproducibility of different techniques used for measuring LA sizes when assessed by observers with different levels of experience have not been evaluated. Our objective was to assess repeatability, reproducibility, and agreement between observers with different levels of experience and experienced cardiologists for three different echocardiographic indices of LA size. Furthermore, our objective was to evaluate the performance of observers with different levels of experience in identifying LAE by two commonly used indices for which prediction intervals from large populations of dogs are reported, i.e., the left atrium to aortic root ratio (LA:Ao) and the left atrial anteroposterior diameter (LA_APD_) [14].

## 2. Materials and Methods

Animals—A power analysis was performed based on acceptable width of the 95% confidence interval (CI) of intraclass correlation coefficient (ICC) [15,16]. The acceptable width of the CI was arbitrarily set at 0.2 and the anticipated value of the ICC was obtained from previously published data on left atrial measurement variability [17] and set at 0.84. A minimum of 35 observations was identified by this analysis. In order to facilitate the selection of an equal number of dogs from three different categories of left atrial size, 36 studies were chosen. An observer not involved with the measurements (AF) performed the study selection, randomization, and blinding as follows. 

The database of echocardiographic reports at the Virginia-Maryland College of Veterinary Medicine was searched for dogs older than 1 year of age that underwent an echocardiographic examination between 1/1/19 and 12/31/19. In order to select studies with a heterogenous distribution of left atrial size, the LA:Ao measurement present in the report was used to subcategorize dogs into three arbitrarily defined groups: LA:Ao < 1.5, 1.5 ≤ LA:Ao ≤ 1.8, LA:Ao > 1.8. Among each group, studies were randomly assigned a number from one to N, where N is the number of studies present in the group. Then, 12 studies per group were randomly selected using an online random number generator, set to generate 12 random numbers between one and N. For each study, the following cineloops were then anonymized using a function embedded in proprietary software (Tomtec Arena, Tomtec Imaging Systems, Unterschleissheim, Germany) and exported as DICOM images, when available: right parasternal four chamber view, right parasternal “inflow-outflow view”, right parasternal short axis view of the heart base at the level of the aorta and left atrium, left parasternal apical “four-chamber view” [18]. Therefore, the observers who performed the measurements were blinded to both the identity of each dog and to their previously reported LA size. Cineloops comprehensive of three consecutive cardiac cycles were selected. Since this was a retrospective study performed on images previously acquired from client-owned animals as part of routine clinical workups, study approval by ethical committee was not necessary.

**Figure 1:**
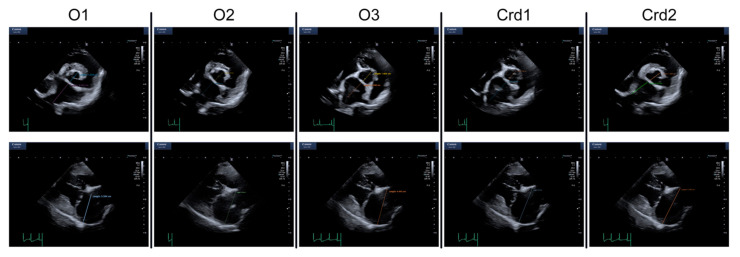
LA:Ao and LA_APD_ performed by different observers. The first row shows LA:Ao measurements of the same dog performed by different observers. The second row shows LA_APD_ measurements of the same dog performed by different observers. O1: veterinary student; O2: first-year cardiology resident; O3: third-year cardiology resident; Crd1: cardiologist 1; Crd2: cardiologist 2.

Echocardiographic measurements—All anonymized cineloops were imported in a software for analysis of medical images (Horos, The Horos Project, Horosproject.org). Five observers with different levels of experience performed the measurements in a blind fashion: a veterinary student with no previous training in echocardiographic measurements (O1), a first-year cardiology resident (O2), a third-year cardiology resident (O3) and two board-certified veterinary cardiologists. Prior to performing the measurements, the veterinary student was instructed to perform literature research on the topic of left atrial measurement in dogs and received only minimal (less than 10 min) verbal directions on how to use the measurement software, but never performed or interpreted any echocardiogram. The first-year cardiology resident had performed and interpreted, at the time of this study, less than 500 echocardiograms. The third-year cardiology resident had performed and interpreted, at the time of this study, approximately 1000 echocardiograms. All variables were measured over three consecutive cycles. Whenever the necessary cineloops were available, the following measurements were performed. The LA:Ao was measured from a right parasternal short axis view in early ventricular diastole defined as the first frame after the end of aortic ejection where the aorta appears with closed aortic cusps, as described by Hansson et al. [19]. The LA_APD_ was measured from right parasternal four-chamber view using a line drawn approximately parallel to the mitral annulus extending from the inner edge of the fossa ovale to the internal reflection of the pericardium in far field parallel to the mitral annulus [14]. The same landmarks were also applied to right parasternal long-axis LV outflow view [18], in order to obtain the left atrial anteroposterior diameter from five-chamber view (LA_APD5CH_). Furthermore, using an apical four-chamber view, the area of the left atrium at the end of ventricular systole (identified as the last frame of mitral valve closure) was traced, excluding the pulmonary veins (LA area) [20]. Given the allometric relationship to body weight previously described, LA_APD_ was divided by BW^0.309^ in order to obtain LAi_APD_ [14]. There was no attempt to standardize which cardiac cycle to measure. However, given the type of cineloops commonly acquired at our institution (i.e., three cardiac cycles, electrocardiogram triggered), most cineloops only contained three measurable cardiac cycles. Although it was not an exclusion criterion, no loop contained pathologic arrhythmias. Examples of LA:Ao and LA_APD_ measurements performed by different observers are presented in Figure 1.

Statistical analysis—All continuous numerical data were analyzed for normality of distribution by visual assessment of normal probability plots. Normally distributed data are presented as mean ± standard deviation, while not normally distributed data are presented as median (25th percentile–75th percentile). The following statistical analyses were performed. Interobserver and intraobserver measurement agreement was assessed by ICC and are presented as ICC (95% CI). For interobserver ICC calculations, the average of the three measurements performed by each observer on each individual was used, and a 2-way average measurement random effect model was chosen [ICC (2,3)]. For intraobserver ICC calculations, the three measurements of each observer were used, and a two-way single measurement mixed effect model was used [ICC (3,1)] [21]. Since well-established canine prediction intervals exist for LA:Ao and LAi_APD_ [14], and all 36 cases had images available for performing these measurements, these two variables were further assessed in terms of their clinical relevance. In order to quantify the degree of agreement between observers with different level of experience and cardiologists, the LA:Ao and LAi_APD_ obtained by the two cardiologists for each case were averaged creating average cardiologists LA:Ao (crdLA:Ao) and average cardiologists LAi_APD_ (crdLAi_APD_), respectively. Bland-Altman plots were then used to assess the agreement between LA:Ao ratio and LAi_APD_ measured by each of the three other observers (O1, O2, O3), and crdLA:Ao and crdLAi_APD_. Furthermore, the measurements of LA:Ao and LAi_APD_ obtained by each of the three less experienced observers were plotted against crdLA:Ao and crdLAi_APD_, respectively. The number of dogs identified as having left atrial enlargement (defined as LA:Ao > 1.7 or LAi_APD_ > 1.65) [14] by the different observers was then evaluated. Furthermore, in order to assess whether a multi-image approach would improve the performance of the observers, we then considered having LAE only dogs that concomitantly fit both above-mentioned criteria. True positives, true negatives, false positives, false negatives, sensitivity, specificity, and positive and negative predictive values were calculated for each observer, for both measurements, and for the combination of the two measurements. Accuracy was calculated as: (true positives + true negatives)/(true positives + true negatives + false positives + false negatives).

## 3. Results

### 3.1. Zoographics

The median age of the 36 randomly selected dogs was 10.7 years (6.3–12.1 years), and the median body weight was 9.1 Kg (5.9–19.6 Kg). Eighteen dogs were female, among which five were spayed, and 18 dogs were males, among which three were neutered. The most represented breed was mixed breed (n: 12), followed by eight Cavalier King Charles Spaniels and two each of the following breeds: Boxer, Dachshund, Maltese. Other represented breeds were Beagle, Boston Terrier, Chihuahua, Cocker Spaniel, French Bulldog, German Shepherd, Golden Retriever, Miniature Schnauzer, Schipperke and Yorkshire Terrier (one each). Twenty-seven dogs were diagnosed with myxomatous valvular degeneration, two with mildly increased aortic velocities, and one each with the following cardiac conditions: arrhythmogenic right ventricular cardiomyopathy, mitral valve dysplasia, pulmonic stenosis, subaortic stenosis, tachycardia-induced cardiomyopathy, ventricular septal defect, and patent ductus arteriosus (echocardiogram obtained one year following ductal occlusion). Five dogs were reported to have previously experienced congestive heart failure (four affected by myxomatous valvular degeneration and one affected by tachycardia-induced cardiomyopathy). Among the dogs affected by myxomatous valvular degeneration without previous evidence of congestive heart failure, 11 were diagnosed with ACVIM Stage B1, 12 with ACVIM Stage B2. Because of the retrospective nature of this study, not all cineloops were present for each study. Particularly, all dogs had a right parasternal four chamber view and a right parasternal short axis view available, while for ten dogs the right parasternal “inflow-outflow” view was not available, and for one dog, the left parasternal apical view was not available.

### 3.2. Intraclass Correlation Coefficients

The interobserver and intraobserver ICC data are presented in Table 1 and Table 2, respectively. Intraobserver ICCs were greater than 0.9 for all the variables. The LA:Ao showed the lowest intraobserver ICC, where O1’s intraobserver ICC was 0.913 (0.855–0.951), while the highest intraobserver ICC was for LA area, where Crd2’s intraobserver ICC was 0.996 (0.993–0.998). Interobserver ICCs were also greater than 0.9 for every variable. The lowest ICC was obtained by LA:Ao measurements (0.968 [0.947–0.982]), while the highest was for LAAPD (0.995 [0.992–0.997]).

### 3.3. Bland–Altman Plots

**Figure 2:**
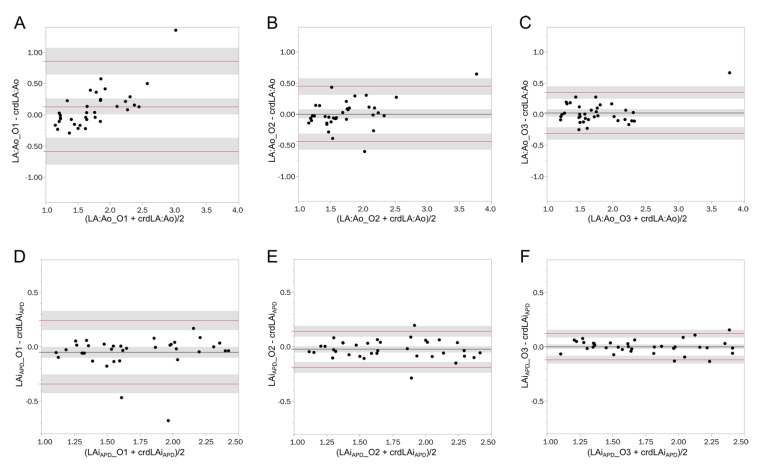
Bland–Altman plots of the left atrial measurements performed by observers with different levels of experience vs. the average of two cardiologists. The black line indicates the mean bias, while the red lines are the 95% limits of agreement. The shaded areas are the 95% CI of the estimates. (**A**–**C**) represents the difference of the measurements of left atrium to aortic root ratio of O1, O2, O3, respectively, plotted against the average left atrium to aortic root ratio of the two cardiologists. (**D**–**F**) represent the difference of the measurements of allometrically scaled left atrial anteroposterior diameter performed by O1, O2, O3, respectively, plotted against the average allometrically scaled left atrial anteroposterior diameter performed by the two cardiologists. crdLA:Ao: average of left atrium to aortic root ratio obtained by the two cardiologists; crdLAi_APD_: average allometrically scaled left atrial anteroposterior diameter obtained by the two cardiologists; LA:Ao_O1: Left atrium to aortic root ratio obtained by O1; LA:Ao_O3: Left atrium to aortic root ratio obtained by O3; LA:Ao_O2: Left atrium to aortic root ratio obtained by O2; LAi_APD__O1: allometrically scaled left atrial anteroposterior diameter obtained by O1; LAi_APD__O3: allometrically scaled left atrial anteroposterior diameter obtained by O3; LAi_APD__O2: allometrically scaled left atrial anteroposterior diameter obtained by O2.

The Bland–Altman plots representing LA:Ao obtained by different observers vs. crdLA:Ao and LAiAPD obtained by different observers against crdLAiAPD are presented in Figure 2. A significant positive bias (0.13 CI: 0.01–0.26) was present for LA:Ao when measured by the observer with least experience (O1). Furthermore, for the same observer, a clear tendency in progressive overestimation of LA:Ao with increasing variable measurement could be noted. No significant bias and no tendency to consistent or progressive under- or overestimation was noted for the other two observers; however, the limits of agreement were narrower with increasing experience. No significant bias and no clear tendency in data distribution was noted in the Bland–Altman plots of LAiAPD, and also for this variable, the limits of agreement were narrower with increasing experience.

### 3.4. Identification of Left Atrial Enlargement

**Figure 3:**
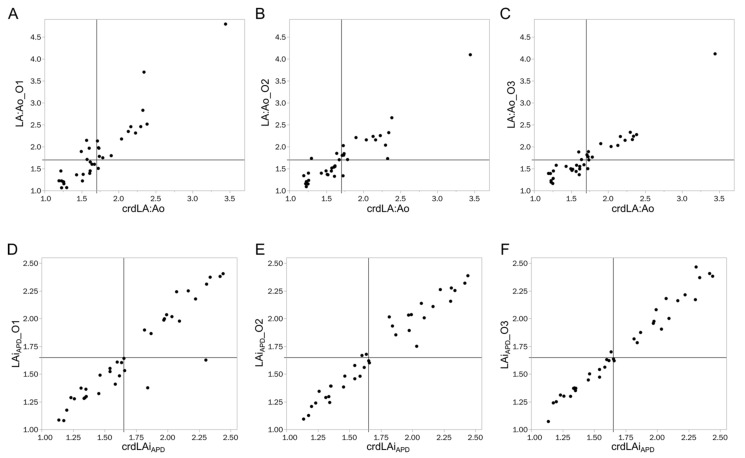
Measurements of left atrium to aortic root ratio and allometrically scaled left atrial anteroposterior diameter obtained from observers with different levels of experience plotted against the average left atrium to aortic root ratio and allometrically scaled left atrial anteroposterior diameter obtained by the two cardiologists. The lines indicate the cutoff for left atrial enlargement (1.7 for left atrium to aortic root ratio, 1.65 for allometrically scaled left atrial anteroposterior diameter). (**A**–**C**) represents the measurements of left atrium to aortic root ratio of O1, O2, O3, respectively, plotted against the average left atrium to aortic root ratio of the two cardiologists. (**D**–**F**) represent the measurements of allometrically scaled left atrial anteroposterior diameter performed by O1, O2, O3, respectively, plotted against the average allometrically scaled left atrial anteroposterior diameter performed by the two cardiologists. crdLA:Ao: average of left atrium to aortic root ratio obtained by the two cardiologists; crdLAi_APD_: average allometrically scaled left atrial anteroposterior diameter obtained by the two cardiologists; LA:Ao_O1: Left atrium to aortic root ratio obtained by O1; LA:Ao_O3: Left atrium to aortic root ratio obtained by O3; LA:Ao_O2: Left atrium to aortic root ratio obtained by O2; LAi_APD__O1: allometrically scaled left atrial anteroposterior diameter obtained by O1; LAi_APD__O3: allometrically scaled left atrial anteroposterior diameter obtained by O3; LAi_APD__O2: allometrically scaled left atrial anteroposterior diameter obtained by O2.

The measurements of LA:Ao and LAiAPD from observers with different levels of experience plotted against crdLA:Ao and crdLAiAPD, respectively, are presented in Figure 3. Data regarding true positives, true negatives, false positives, false negatives, positive predictive value, negative predictive value, and accuracy for LA:Ao, LAiAPD, and the combination of both variables to identify LAE are presented in Table 3. In summary, neither LA:Ao nor LAiAPD had 100% sensitivity or specificity for any observer. Notably, the overall accuracy increased with the level of experience for both measurements, and LAiAPD had higher accuracy than LA:Ao for every observer. Nonetheless, the least experienced observer had more false negatives, and, therefore, a lower negative predictive value when using LAiAPD, compared to LA:Ao. When using a combined approach (defining dogs as having LAE only when both LA:Ao and LAiAPD were above the upper limit of prediction intervals), accuracy and negative predictive values for the two least experienced observers was the highest.

## 4. Discussion

In this study we assessed the repeatability, reproducibility, and agreement with experienced cardiologists of different echocardiographic indices of LA size between observers with different levels of experience. Our findings indicate that all the variables investigated have excellent repeatability and reproducibility, even when measured by observers with very limited experience. While data about repeatability and reproducibility are not available for LA_APD5CH_ and LA area, in our study we found similar intraobserver ICCs for LA:Ao and LA_APD_, to what recently reported in another study, where measurements were performed by a trained diagnostic imaging resident on a selected group of healthy dogs [17]. The interobserver ICCs that we found in this study for LA:Ao and LA_APD_, are instead slightly higher than previously reported in both healthy dogs and dogs affected by myxomatous valvular degeneration, even though all the observers in these studies were reported to have received some training [17,22]. This is explained by the different forms of interobserver ICC. In fact, in this study we used a “*average measures*” form of ICC (referred to as [ICC 2,3], where three indicates that the average of three measurements was used for each observer), while in the cited studies a “*single measures*” approach was used. *Average measures* forms result in higher ICCs. Nonetheless, since often in clinical practice the average of three measurements is used to direct clinical decisions, the results of this study are expected to apply [21].

When compared to the criterion standard comprised by the average of two cardiologists, we found that the least experienced observer is biased towards overestimation of left atrial sizes when using LA:Ao, and this tendency increases with increasing left atrial sizes. However, it must also be noted that the interpretation of the mean bias requires caution when a proportional bias is also present, since both over- and underestimation may occur for extreme measurements. This may result in inappropriate false diagnosis of enlarged left atrium (4/36 (11%) dogs were false positives when O1 used LA:Ao to evaluate the left atrium in the population of this study), which could potentially result in inappropriate treatment decisions. Conversely, when using LAi_APD_, the same observer missed LAE in 3/36 (8%), which could as well result in significant errors in decision-making. Not surprisingly, we found that the agreement with cardiologists and the overall accuracy in identification of LAE using LA:Ao or LAi_APD_ increased with the level of experience. Furthermore, combining both variables for identifying LAE greatly increased accuracy although, obviously, this produces a more stringent criterion.

This study has several limitations. First and foremost, the fact that all images were acquired by either residents in training under the direct supervision of a cardiologist, or by cardiologists. Therefore, the results of this study cannot be applied to a real-life clinical scenario, in which different observers are introducing variability both in the phase of acquisition and measurement of the images. Similarly, the observers were blinded to signalment and clinical presentation of the dogs, which is another factor that can potentially introduce a bias in both image acquisition and interpretation phases. This study intentionally aimed to evaluate only the performance of different variables for measuring the left atrium by observers with different level of experience. Further studies to evaluate the variability introduced by observers with different levels of experience in both acquiring and measuring canine left atria are warranted. Lastly, there was only one observer for each level of experience evaluated; therefore, generalizability of these results to a broader population of inexperienced observers requires caution.

## 5. Conclusions

In conclusion, LA:Ao, LA_APD_, LA_APD5CH_, and LA area have excellent repeatability and reproducibility even when used by the least experienced observers. The accuracy of LA:Ao and LAi_APD_ in identifying left atrial enlargement increases with the level of experience of the observer, and by combining the two variables.

## Figures and Tables

**Table 1:** Intraobserver Intraclass Correlation Coefficients (95% CI). Ao_SAX_: aortic diameter obtained from parasternal short axis view at the level of the heart base; LA area: left atrial area obtained from right parasternal apical view; LA_APD_: left atrial anteroposterior diameter obtained from right parasternal four chamber view; LA_APD5CH_: left atrial anteroposterior diameter obtained from right parasternal “inflow-outflow” view; LA_sax_: left atrial diameter obtained from parasternal short axis view at the level of the heart base.

		Observer			
	O1	O2	O3	Crd1	Crd2
Variable					
Ao_sax_	0.946 (0.909–0.970)	0.976 (0.959–0.987)	0.976 (0.959–0.987)	0.967 (0.943–0.982)	0.986 (0.976–0.992)
LA_sax_	0.984 (0.973–0.991)	0.989 (0.981–0.994)	0.971 (0.951–0.984)	0.972 (0.952–0.985)	0.989 (0.980–0.994)
LA:Ao	0.913 (0.855–0.951)	0.968 (0.945–0.982)	0.929 (0.881–0.961)	0.925 (0.874–0.958)	0.971 (0.950–0.984)
LA_APD_	0.986 (0.976–0.992)	0.984 (0.973–0.991)	0.990 (0.982–0.994)	0.985 (0.974–0.992)	0.994 (0.989–0.997)
LA_APD5CH_	0.952 (0.912–0.977)	0.978 (0.959–0.989)	0.977 (0.957–0.989)	0.959 (0.924–0.980)	0.983 (0.966–0.992)
LA area	0.994 (0.989–0.997)	0.996 (0.993–0.998)	0.992 (0.987–0.996)	0.988 (0.980–0.994)	0.996 (0.993–0.998)

**Table 2:** Interobserver Intraclass Correlation Coefficients (95% CI). Ao_SAX_: aortic diameter obtained from parasternal short axis view at the level of the heart base; LA area: left atrial area obtained from right parasternal apical view; LA_APD_: left atrial anteroposterior diameter obtained from right parasternal four chamber view; LA_APD5CH_: left atrial anteroposterior diameter obtained from right parasternal “inflow-outflow” view; LA_sax_: left atrial diameter obtained from parasternal short axis view at the level of the heart base.

Variable	ICC (95% CI)
Ao_sax_	0.989 (0.979–0.994)
LA_sax_	0.985 (0.975–0.991)
LA:Ao	0.968 (0.947–0.982)
LA_APD_	0.995 (0.992–0.997)
LA_APD5CH_	0.990 (0.982–0.995)
LA area	0.984 (0.975–0.991)

**Table 3:** Accuracy in identification of left atrial enlargement by different observers, using left atrium to aortic root ratio, allometrically scaled left atrial anteroposterior diameter, or combining both variables. FN: false negatives; FP: false positives; LA:Ao: left atrium to aortic root ratio; LAi_APD_: allometrically scaled left atrial anteroposterior diameter; NPV: negative predictive value; PPV: positive predictive value; Se: sensitivity; Sp: specificity; TN: true negatives; TP: true positives.

	Observer	TP	TN	FP	FN	Se	Sp	PPV	NPV	Accuracy
LA:Ao	O1	15	16	4	1	94%	94%	79%	94%	86%
	O2	14	18	2	2	88%	90%	88%	90%	89%
	O3	14	19	1	2	88%	90%	93%	90%	92%
LAi_APD_	O1	14	19	0	3	82%	86%	100%	86%	92%
	O2	16	17	2	1	94%	94%	89%	94%	92%
	O3	16	18	1	1	94%	95%	94%	95%	94%
Both	O1	10	24	1	1	91%	96%	91%	96%	94%
	O2	10	24	1	1	91%	96%	91%	96%	94%
	O3	9	25	0	2	82%	93%	100%	93%	94%

## Data Availability

The data and models reported within this study are available from the corresponding author upon reasonable request.
